# Association of microtubules and axonal RNA transferred from myelinating Schwann cells in rat sciatic nerve

**DOI:** 10.1371/journal.pone.0233651

**Published:** 2020-05-29

**Authors:** Lucía Canclini, Joaquina Farias, Andrés Di Paolo, José R. Sotelo-Silveira, Gustavo Folle, Alejandra Kun, José R. Sotelo

**Affiliations:** 1 Departamento de Genética, Instituto de Investigaciones Biológicas Clemente Estable, Montevideo, Uruguay; 2 Departamento de Proteínas y Ácidos Nucleicos, Instituto de Investigaciones Biológicas Clemente Estable, Montevideo, Uruguay; 3 Departamento de Genómica, Instituto de Investigaciones Biológicas Clemente Estable, Montevideo, Uruguay; 4 Departamento de Biología Celular y Molecular, Facultad de Ciencias, UdelaR, Montevideo, Uruguay; 5 Unidad asociada a Sección Bioquímica, Facultad de Ciencias, UdelaR, Montevideo, Uruguay; Universitat Wien, AUSTRIA

## Abstract

Transference of RNAs and ribosomes from Schwann cell-to-axon was demonstrated in normal and regenerating peripheral nerves. Previously, we have shown that RNAs transfer is dependent on F-actin cytoskeleton and Myosin Va. Here, we explored the contribution of microtubules to newly synthesized RNAs transport from Schwann cell nuclei up to nodal microvilli in sciatic nerves. Results using immunohistochemistry and quantitative confocal FRET analysis indicate that Schwann cell-derived RNAs co-localize with microtubules in Schwann cell cytoplasm. Additionally, transport of Schwann cell-derived RNAs is nocodazole and colchicine sensitive demonstrating its dependence on microtubule network integrity. Moreover, mRNAs codifying neuron-specific proteins are among Schwann cell newly synthesized RNAs population, and some of them are associated with KIF1B and KIF5B microtubules-based motors.

## Introduction

Neuronal soma and glial cells in myelinated fibers sustain axonal proteostasis. Local protein synthesis contribution to axonal proteostasis has been demonstrated and play roles in development, maintenance, and neuronal function (for extensive and recent review see [1,2]). From the classical point of view, axonal RNAs are conveyed from the neuronal soma by axoplasmic transport, a mechanism that has been studied exhaustively (recently reviewed in [3]). In addition, we have demonstrated that RNAs found in peripheral axons can be locally transferred from Schwann cells in normal and regenerating conditions [4,5]. The transferred RNA pool includes molecules transcribed by RNA Polymerase II [5] and rRNA/ribosomes [5–9]. We also determined that Schwann cell-to-axon RNA transfer occurs mainly at nodes of Ranvier in an actin cytoskeleton dependent fashion [5]. Transferred RNA was found associated with the actin-based molecular motor Myosin Va, at nodes of Ranvier [4]. Furthermore, null mutant mice for Myosin Va showed RNA accumulation in Schwann cell nodal microvilli and RNA absence in axons [5]. These results clearly demonstrated that glia-to-axon transfer across the membranes of both cells is dependent on F-actin, but how RNA reached Schwann cell nodal microvilli has not been elucidated until now.

RNA transport to specific subcellular sites in the nervous system mainly occurs along microtubules involving kinesin molecular motors (for extensive and recent review see [10]). Several mRNAs have been identified enriched in oligodendrocytes processes [11,12], where they could be delivered by microtubules-based transport [13]. It has been determined that myelin basic protein (*MBP*) mRNA is selectively transported to oligodendrocyte processes in a kinesin-3 (KIF1B) dependent manner [14]. On the other hand, mRNAs in neurons were found associated with kinesin-1 heavy chains (KIF5) [15,16].

In this work, we explored the contribution of microtubules to the RNA transport from perinuclear areas of Schwann cell to axon through nodal microvilli. Based in a pharmacological approach using nocodazole and colchicine we determined that the RNA transport within Schwann cell is dependent on microtubules. In addition, we explored the association of KIF1B and KIF5B with RNA transcribed in Schwann cells.

## Materials and methods

### Surgery

Male CD Sprague Dawley IGS rats (Charles River Laboratory, USA) were purchased from the Unit of Experimental Reactives and Biomodels of the Faculty of Medicine, University of the Republic (URBE-FMed-UdelaR, Montevideo, Uruguay). All experiments were performed on 3 to 6 months-old adult rats. Animal procedures were performed in accordance with the Animal Experimentation National Commission (CNEA) guidelines together with the Law of Uruguay regarding the care and use of experimental animals and approved by the appropriate state committees for animal welfare. The specific protocol was approved by the CNEA-IIBCE (Protocol Sotelo-013/09/2011). Sciatic nerve injury was performed as previously described [4]. Briefly, adult rats were anesthetized with 5/10 mg/kg ketamine/xylacine, and sciatic nerves were exposed by a mid-thigh incision. Sciatic nerves were transected and the incision closed thereafter. Proximal nerve stumps were isolated at 18 hours post-surgery. Injured sciatic nerves were used in all experiments. Rats were sacrificed by overdose of anesthetic (10/20 mg/kg ketamine/xylacine) and euthanasia was confirmed by decapitation.

### RNA labeling

For RNA labeling, isolated proximal nerve stumps were treated as was previously reported [4,5,17]. Briefly, proximal nerve stumps were incubated for 6 hours (37°C, 5% CO_2_) in freshly prepared Neurobasal medium (Gibco, Thermo Fisher Scientific, Germany) containing 5 mg mL^-1^ of 5-Bromouridine (BrU, AlfaAesar, Tewksbury, MA, USA), which is co-transcriptionally incorporated into the newly synthesized RNA (BrU-RNA). For microtubules disruption experiments, nocodazole and colchicine were used in accordance with the literature [18–21]. Nocodazole (0.4 μM and 4 μM, Sigma-Aldrich, Saint Louis, MO, USA) or colchicine (5 μM, Sigma-Aldrich, Saint Louis, MO, USA) were added to the BrU-containing media during RNA labeling.

### Pre-immunohistochemistry treatments

After RNA labeling, proximal nerve stumps were treated to remove monomeric tubulin according to Khawaja et al. [22] with a few modifications. Nerve segments were incubated 10 min in PEM buffer (85 mM PIPES, 10 mM EGTA, 1 mM MgCl_2_, pH 6.94) at 25°C, and then extracted for 1 hour with freshly prepared 0.5% Triton X-100 (Sigma-Aldrich, Saint Louis, MO, USA) in PEM buffer, at 25°C. Tubulin monomer extraction of nerve stumps exposed to colchicine or nocodazole was performed in buffer supplemented with each drug in order to prevent repolymerization of microtubules. After tubulin extraction, nerve stumps were rinsed once for 5 min in PEM buffer and then fixed.

For RNAse treatment, nerve stumps were incubated in 10 mg mL^-1^ for 1 h, at 37°C and then fixed [5].

### Immunohistochemistry

Tissue fixation for microtubules visualization can be performed using either glutaraldehyde, methanol or formaldehyde (for an excellent guide see https://mitchison.hms.harvard.edu/files/mitchisonlab/files/fluorescence_procedures_for_the_actin_and_tubulin_cytoskeleton_in_fixed_cells.pdf). Glutaraldehyde is the best fixation method for microtubule preservation but is incompatible with co-staining procedures. Although methanol is the second fixation condition of choice for microtubular preservation, it is incompatible with myelinated fibers structure preservation. Therefore, nerve stumps fixation was performed in freshly prepared 3% paraformaldehyde for 30 min. Nerve stumps were treated for 1 hour at 37°C with 0.225 mg mL^-1^ collagenase (Sigma-Aldrich, Saint Louis, MO, USA) in PHM buffer (60 mM PIPES, 25 mM HEPES, 2 mM MgCl_2_, pH 7.4) and 5 mM CaCl_2_. Nerve fibers were released from epineurium with #5 forceps.

The incubation buffer for all following immunohistochemistry steps was 0.1% BSA (Sigma-Aldrich, Saint Louis, MO, USA) and 2% glycine in PHEM buffer (60 mM PIPES, 25 mM HEPES, 10 mM EGTA, 2 mM MgCl_2_, pH 7.4). Nerve fibers were prepared for immunohistochemistry by blocking in 10% normal goat serum (Life Technologies, Thermo Fisher Scientific, USA) for 30 min, and then incubated with anti-BrdU (also reactive to BrU, Cat. No. 11170376001, Roche Diagnostics GmbH, Mannheim, Germany, 5 μg mL^-1^), anti-β-tubulin (Cat. No. ab6049, Abcam, Cambridge, MA, USA, 1:200), anti-vimentin (Cat. No. ab92547, Abcam, Cambridge, MA, USA, 1:250), anti-Myelin Associated Glycoprotein–MAG- (Cat. No. MAB5567, Chemicon, Burlington, MA, USA, 3 μg mL^-1^), anti-Caspr2 (Cat. No. ab33994, Abcam, Cambridge, MA, USA, 1:100), anti-neurofilament light chain–NEF-L- (Cat. No. 2835, Cell Signaling Technology, Danvers, MA, USA, 1:100), anti-KIF1B (Cat. No. ab69614, Abcam, Cambridge, MA, USA, 1:200), anti-KIF5B (Cat. No. ab167429, Abcam, Cambridge, MA, USA, 1:200) or anti-KIF1A (Cat. No. PAB20294, Abnova, Taipei, Taiwan, 1:200), overnight at 4°C. Nerve fibers were washed 3 times 10 min each. Secondary antibodies (goat anti-mouse Alexa Fluor 488 -Cat. No. A11029-, goat anti-mouse Alexa Fluor 555 -Cat. No. A21424-, goat anti-rabbit Alexa Fluor 488 -Cat. No. A11034- and goat anti-rabbit Alexa Fluor 555 -Cat. No. A21429-, all from Invitrogen, Thermo Fisher Scientific, USA, all 1:1000) were incubated for 2 hours at room temperature. F-actin was detected using Alexa 633-phalloidin (Invitrogen, Thermo Fisher Scientific, USA, 1:150) added together with secondary antibodies and nuclei were revealed through DAPI staining (Invitrogen, Thermo Fisher Scientific, USA, 0.3 nM). Fibers were then washed 3 times, 10 min each. Finally, individual fibers were teased and mounted in ProLong Gold Antifade (Invitrogen, Thermo Fisher Scientific, USA) for immunohistochemistry and PFRET analysis or mounted in glycerol for APb-FRET analysis.

### Microscopic analysis

For confocal microscopy studies a Zeiss LSM800 furnished with a 63x oil immersion objective (NA: 1.4, Carl Zeiss International, Oberkochen, Germany) was used.

### FRET analysis

Secondary goat anti-rabbit and anti-mouse Alexa labelled antibodies were used in all FRET experiments. Alexa-Fluor 488 fluorophore was used as donor and Alexa-Fluor 555 was used as acceptor for all the FRET experiments presented here. Sensitized emission quantitative Förster resonance energy transfer (PFRET) analysis of immunolabeled nerve fibers was performed as previously described [4]. Images were collected using single-labeled donor and acceptor samples and double-labeled samples treated for immunohistochemistry as described above. Four single-label donor reference images (donor excitation in both donor and acceptor channels); four single-label acceptor reference images (donor and acceptor excitation, both in the acceptor channel); and six to ten double-label images (donor excitation in donor and acceptor channels, acceptor excitation in acceptor channel) were taken. Control samples incubated without specific antibodies and incubated only with secondary antibodies were also included. Images of control samples were collected with donor and acceptor excitation lasers in both channels, including images in the donor channel at acceptor excitation to check for back-bleedthrough. All images were acquired at identical, previously optimized settings, using the same photomultiplier values (800V) in donor and acceptor channels and low laser power (0.2% for both 488 nm and 561 nm diode laser). Quantitative FRET analysis was performed using the PFRET plugin (pFRET analysis option) for ImageJ (Keck Center for Cellular Imaging, University of Virginia, Charlottesville, VA22903) [23–27]. Background noise was subtracted using control images to calculate the gray-level value of background. Regions of interest (ROIs) were created automatically by the PFRET software. Image depth (8-bit), Förster distance R0 value (67.5 Å, for Alexa-Fluor 488 and Alexa-Fluor 555 FRET pair), quantum yields of donor (Qd: 0.91 for Alexa-fluor 488) and acceptor (Qa: 0.1 for Alexa-fluor 555) values were inserted in the PFRET software. To eliminate outliers (data due to random interactions), the same restrictions (A, D and uFRET > 10; PFRET and E% > 5; uDA < 6) were used in all FRET experiments performed with the different pair partners [4]. Additionally, an assay based on the relationship between FRET efficiency, E% [25,28] and acceptor levels was used to separate random and specific interactions that can take place in crowded cellular environments [26,29,30]. In a random situation, the likelihood of an acceptor colocalizing with a given donor is positively correlated with acceptor levels and leads to an increase in E%. Conversely, in a clustered situation E% is independent of acceptor levels (or negatively dependent).

Acceptor-photobleaching Förster resonance energy transfer (APb-FRET) analysis of immunolabeled nerve fibers was also performed [31]. Images were collected using double-labeled samples treated for immunohistochemistry as described above. Single-labeled donor sample was included to control the amount of donor photobleaching during image acquisition and acceptor bleaching procedure. Donor and acceptor images (before Pb-donor and acceptor images) of selected areas were collected at the same PTM value for both channels (800V) and low laser power (0.2% for both 488 nm and 561 nm diode laser). The zoom was changed to 8x, which resulted in the capture of only the centrally located region. Acceptor photobleaching was achieved after 5 minutes of continuous 561 nm laser scanning at 100% power level. The zoom was changed back to 1x and new donor and acceptor images (after Pb-donor and acceptor images) were acquired at initial conditions (800V PMT-value and 0.2% laser power for both channels). APb-FRET analysis was performed using the PFRET plugin (apFRET analysis option) for ImageJ (Keck Center for Cellular Imaging, University of Virginia, Charlottesville, VA22903) [23,26]. Background noise was subtracted using control images to calculate the gray-level value of background. Regions of interest (ROIs) were created automatically by the PFRET software. Image depth (8-bit), and Förster distance R0 value (67.5 Å, for Alexa-Fluor 488 and Alexa-Fluor 555 FRET pair) were inserted in the PFRET software.

### BrU-RNA sequencing

For BrU-labeled RNA (BrU-RNA) sequencing, 10 proximal nerve stumps (3–5 mm) previously incubated in BrU-Neurobasal medium for 6 hours, were homogenized in TRIzol (Invitrogen, Thermo Fisher Scientific, USA). Purified RNA (20 μg) were immunoprecipitated, using 10 μg of anti-BrdU (Abbiotec, San Diego, CA, USA), pre-coupled to 50 μL of Dynabeads Protein G (Life Technologies, Thermo Fisher Scientific, USA) in RSB buffer (10 mM Tris-Cl pH 7.4, 100 mM NaCl, 2.5 mM MgCl_2_, 0.4% Triton X-100, 80 U RNase OUT -Invitrogen, Thermo Fisher Scientific, USA- and 2.5 μg of yeast transfer RNA -Sigma-Aldrich, St. Louis, MO, USA). The mixture was incubated at 4°C for 1 hour under rotation. Dynabeads were washed 3 times with 1 mL RSB buffer, and bound RNA was purified with RNAqueous-MicroTotal RNA Isolation kit (Life Technologies, Thermo Fisher Scientific, USA). Control for unspecific immunoprecipitation was performed using 10 proximal nerve stumps previously incubated in Neurobasal medium without BrU.

BrU-RNA sequencing was performed with the Ion Torrent technology (Life Technologies, Thermo Fisher Scientific, USA). Quantity and quality of RNA were evaluated using NanoDrop2000 spectrophotometer (Thermo Fisher Scientific, USA) as well as Bioanalyzer 2100 (Agilent Technologies, CA, USA). For library preparation 15 μL of BrU-RNA immunoprecipitated sample (50 ng) and the same volume of control sample (containing no measurable RNA) were used. An External RNA Controls Consortium (ERCC) RNA spike-in control mix (Ambion, Life Technologies, Thermo Fisher Scientific, USA) was added to both total RNA inputs before ribosomal RNA depletion using the Low Input RiboMinus^TM^ Eukaryotic System v2 (Ambion, Life Technologies, Thermo Fisher Scientific, USA). Preparation of a whole-transcriptome RNA library from purified RNA was performed by means of an Ion Total RNA-Seq kit v2 (Life Technologies, Thermo Fisher Scientific, USA). Template was prepared with the OneTouch Ion^TM^ Template Kit (Life Technologies, Thermo Fisher Scientific, USA), and then sequenced on 318 semiconductor chip using the Ion PGM^TM^ 200 Sequencing Kit and PGM chemistry (Life Technologies, Thermo Fisher Scientific, USA), following the manufacturer’s instructions.

Sequencing reads were mapped to rat reference genome (*Rattus norvegicus*, Rnor_6.0.95) with HISAT2 v2.1.0 [32]. The resulting files were sorted and converted into BAM files using SAMtools [33]. Alignments were then elaborated by StringTie v1.3.3 [34], which assembled and quantified the transcripts in each sample. Sequencing reads counts were calculated using the Python script (prepDE.py) provided with StringTie. Levels of gene expression were assessed and normalized to transcripts per million (TPM). Genes with a TPM ≥1 were considered as present in the sample.

### Anti-KIF immunoprecipitation and quantitative PCR

For ribonucleoprotein complexes immunoprecipitation experiments, fibers of 10 proximal nerve stumps (3–5 mm) were released from epineurium and homogenized in freshly prepared homogenization buffer (HB, 1 mg of tissue: 4 μL HB, 50 mM Tris-Cl pH 7.4, 100 mM NaCl, 1 mM EDTA, 0.4% Triton X-100 and 1x SigmaFast Protease Inhibitor Cocktail -Sigma-Aldrich, St. Louis. MO, USA). After centrifugation at 10,000 xg for 10 min at 4°C the supernatants were collected and mixed with either 10 μg of anti-KIF1B (No. ab69614, Abcam, Cambridge, MA, USA), or 10 μg of anti-KIF5B (No. ab167429, Abcam, Cambridge, MA, USA) or 10 μg of anti-BrdU (No. 250563, Abbiotec, San Diego, CA, USA, unspecific immunoprecipitation negative control). Mixtures were incubated at 4°C for 1 hour under rotation. Immediately, 50 μL of Dynabeads Protein G (Life Technologies, Thermo Fisher Scientific, USA) were added, and mixtures were rotated for an additional hour. Dynabeads were washed 3 times with 1 mL HB, and RNA was eluted and purified with RNAqueous-MicroTotal RNA Isolation kit (Life Technologies, Thermo Fisher Scientific, USA).

Purified RNA was reverse transcribed into cDNA using oligo-dT primer and SuperScript III Reverse Transcriptase (Invitrogen, Thermo Fisher Scientific, USA). cDNA was used to prepare triplicate reactions for qPCR according to SYBR Green Universal Master Mix (Applied Biosystems, Thermo Fisher Scientific, USA) manufacturer’s instructions and run on a CFX96 Touch Real-Time PCR Detection System (Bio-Rad, Hercules, CA, USA) using the following PCR conditions: denaturation for 15 s at 95°C; annealing and extension for 1 min at 60°C. The levels for each condition were corrected with their own input. The following primers were used for qPCR: *Nef-L*, 5′-CCGGTTCTTCTCTCTAGGTCCC-3′ and 5′-GTAGGAGGTCGAAAAGTACGGC-3′; *Kif1a*, 5′-CGAGGTCTTCGGGCACTA-3′ and 5′-CATGACCCTGGGGAAGTG-3′; *Kcna1*, 5′-ACCAGGCAAGCAATCAAAAG-3′ and 5′-AAAGCATTCAAAGACTACGGTTTT-3′; *Sptan*, 5′-TTGATGTCCGATCTCAGTGC-3′ and 5′-TTGCCAGTCTCATCATCCAT-3′; *Sptbn*, 5′-AGGCAGCGTCTTGAGATGA-3′ and 5′-CCTGAGACAGCAATAGCACCT-3′; *MBP*, 5′-ACGGACACCCTTTCAAGTTCAC-3′ and 5′-GTGCCTGTCTATCCGCAGTG-3′.

### Quantification and statistical analysis

Experiments were performed in at least three independent biological replicates. For microscopy quantitative analyses, at least 10 fiber images were taken. Images were post-processed with ImageJ (http://imagej.nih.gov/ij/). BrU-RNA fluorescence intensity was measured along a line drawn on Schwann cell cytoplasm, or axoplasm and fluorescence intensities were averaged every 2.5 μm along the Schwann cell cytoplasm or axoplasm, according to Sotelo et al. [5]. For FRET analysis, data generated was processed using EXCEL. Statistical analyses used in this study included two-way ANOVA and Pearson correlation test. Statistical significance is defined as, * p < 0.05, ** p < 0.01, *** p < 0.001 and **** p < 0.0001. Pearson correlation coefficient of <0.1 indicates a negligible relationship.

## Results

**Fig 1:**
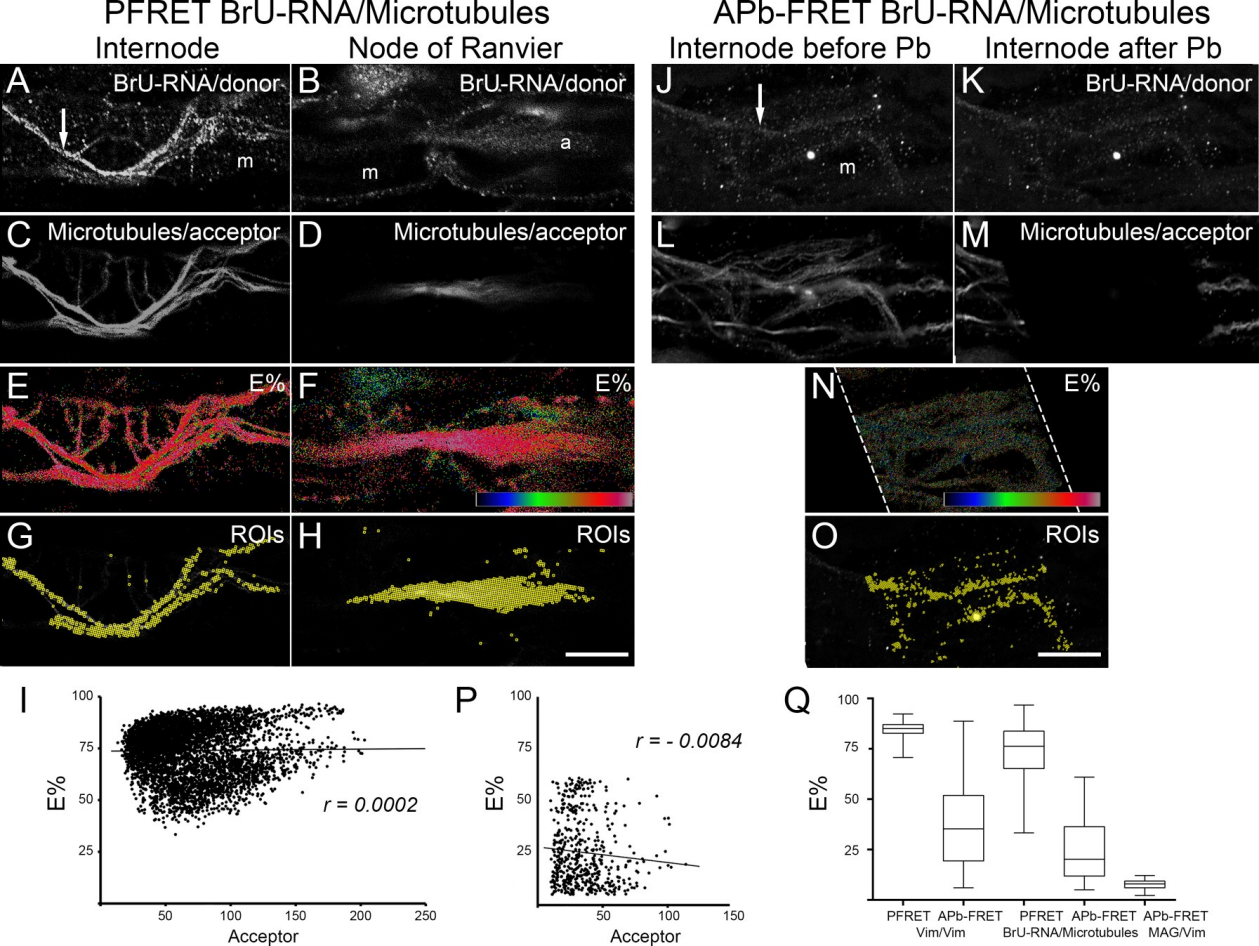
Confocal PFRET and APb-FRET analysis of co-localization between BrU-RNA and microtubules in proximal nerve stumps. Representative single focal planes of sciatic nerve fibers at the internode and node of Ranvier are shown. Fibers were labeled to visualize newly synthesized RNA (BrU-RNA) as donor (A, B, J, K) and microtubules as acceptor (C, D, L, M). For APb-FRET analysis donor and acceptor channels are showed before (J and L, respectively) and after (K and M, respectively) photobleaching (Pb). FRET efficiency (E%, rainbow LUT 0% -black- to 100% -white) at each pixel is indicated (E, F, N). Position on Schwann cell internodal cytoplasm and axoplasm of ROIs having specific FRET signal is also depicted (G, H, O). E% versus acceptor level is plotted (I, P) for ROIs having specific FRET signal (*r = 0*.*0002* for PFRET analysis–I- or R = -0.0084 for APb-FRET analysis). E% distribution obtained with BrU-RNA and microtubules PFRET and APb-FRET analysis are compared (Q) with those obtained for PFRET and/or APb-FRET analysis of positive FRET control (Vim/Vim) and negative FRET control (MAG/Vim). Dotted white line in (N) delineate the photobleached zone. Bar size: 20 μm. m: myelin. a: axon. Arrow: Cajal band.

In this work, we analyzed the localization of BrU-RNA in relation with microtubules by confocal immunomicroscopy (Fig 1). BrU-RNA signal at the internode was found in abaxonal Schwann cell cytoplasm (Fig 1A–1J), particularly at the cytoplasmic channels known as Cajal bands (S1A Fig, arrows). At node of Ranvier, Schwann cell cytoplasm is organized in actin-rich paranodal loops and nodal microvilli (S1B Fig, asterisk and arrow, respectively) in which BrU-RNA signal was detected (Fig 1B). Axoplasm at node of Ranvier (Fig 1B) was also positive for BrU-RNA signal. BrU-RNA is completely absent in myelin domain (Fig 1A and 1B). To assess the specificity of BrU labeling into RNA we performed a series of negative control experiments (S1C–S1F Fig), namely: a) in the absence of BrU (S1D Fig); b) BrU labeling without primary anti-BrU antibody (S1E Fig); c) BrU labeling and subsequent treatment with ribonuclease A before fixation (S1F Fig). All negative controls showed little or no BrU signal of Schwann cells or axons.

**Fig 2:**
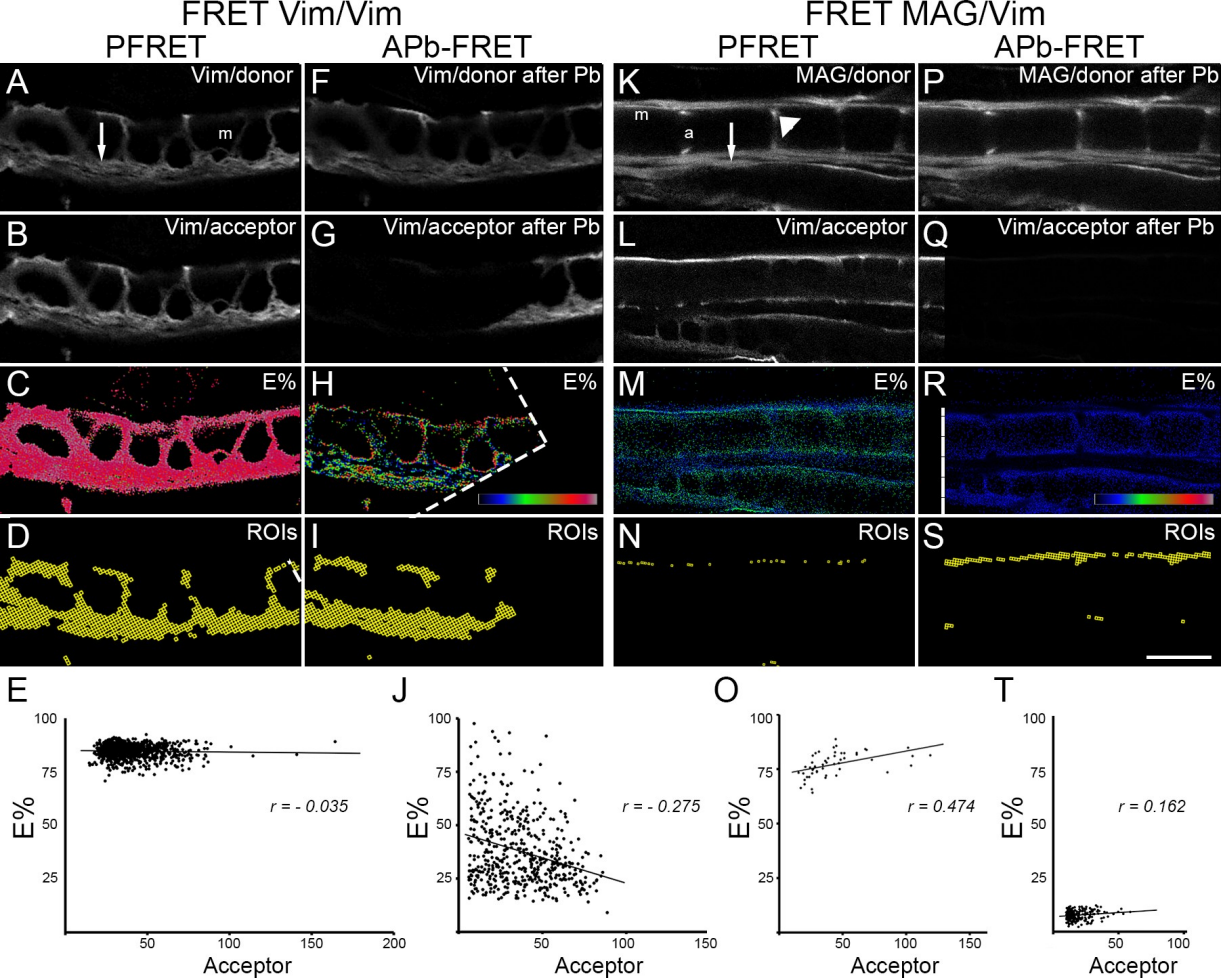
Confocal PFRET and APb-FRET analysis of positive and negative controls. Representative single focal planes of sciatic nerve fibers at the internode are shown. (A-J) Positive FRET analysis control: Fibers were labeled to visualize the same primary antibody (rabbit anti-vimentin) using both 488-Alexa conjugate as donor (A, Vim/donor) and 555-Alexa conjugated anti-rabbit antibody as acceptor (B, Vim/acceptor). After imaging for PFRET analysis (A, B), acceptor was bleached (G, Vim/acceptor after Pb) and a new donor image was taken (F, Vim/donor after Pb). Note that A and B images were used for PFRET analysis and for APb-FRET analysis as “donor and acceptor before Pb images”. FRET efficiency (E%, rainbow LUT 0% -black- to 100% -white) at each pixel is indicated (C, H). Position of ROIs having specific PFRET signal is also depicted (D, I). E% versus acceptor level is plotted (E, J) for ROIs having specific FRET signal (*r = - 0*.*035* for PFRET analysis–E- or R = -0.275 for APb-FRET analysis–J-). (K-T) Negative FRET analysis control: Fibers were labeled to visualize MAG as donor (K, MAG/donor) and vimentin as acceptor (L, Vim/acceptor). After imaging for PFRET analysis (K, L), acceptor was bleached (Q, Vim/acceptor after Pb) and a new donor image was taken (P, MAG/donor after Pb). Note that K and L images were used for PFRET analysis and for APb-FRET analysis as “donor and acceptor before Pb images”. FRET efficiency (E%, rainbow LUT 0% -black- to 100% -white) at each pixel is indicated (M, R). Position of ROIs obtained after analysis is also depicted (N, S). E% versus acceptor level is plotted (O, T) showing random FRET signal (*r = 0*.*474*) for PFRET analysis and E% baseline distribution for APb-FRET analysis. Dotted white line in (H, R) delineate the photobleached zone. Bar size: 20 μm. m: myelin. a: axon. Arrow: Cajal band. Arrowhead: Schmidt-Lanterman Incisure.

Microtubule signal was found in Schwann cell cytoplasm at internode (Fig 1C–1L) and in axoplasm (Fig 1D). Since microtubules signal was distributed throughout the cytoplasm, instead of conventional confocal microscopy, confocal FRET analysis was preferred to assess the co-localization of BrU-RNA and microtubules. This analysis was performed by two independent FRET approaches, sensitized emission FRET (PFRET) and acceptor photobleaching FRET (APb-FRET). In order to assess the behavior of both FRET approaches in our system, we performed relevant FRET controls, including positive and negative controls (Fig 2). Donor and acceptor labeled secondary anti-antibodies recognizing vimentin cytoskeleton was chosen as a positive FRET control (PFRET, Fig 2A–2E, and APb-FRET, Fig 2F and 2J). For both FRET approaches, the relationships of E% with acceptor levels correspond to a clustered FRET interaction. For PFRET analysis, E% was independent on acceptor level (*r = -0*.*035*, Fig 2E) ranging from 70 to 92%, with a mean ± SD = 84 ± 3%. For the APb-FRET analysis, E% was weakly negatively related to acceptor levels (*r = - 0*.*275*, Fig 2J) ranging from 6 to 88%, with a mean ± SD = 36 ± 20%. Positive FRET control experiment results indicates that E% means between both FRET approaches evaluated have 48 points (84 versus 36%) of difference, but E% values of PFRET analysis fall within the range showed by E% values from APb-FRET analysis, with standard deviation ranges (error bars) overlapping, as was also showed by other authors’ PFRET-APb-FRET analyses comparisons [26,35]. For a negative control, two proteins (Myelin Associated Glycoprotein–MAG- and vimentin) expressed in the same cellular compartment (Schwann cell cytoplasm including abaxonal cytoplasm and Schmidt-Lanterman incisures) but that do not interact [36] were choose. MAG and vimentin co-localization was evaluated by PFRET (Fig 2K–2O) and APb-FRET (Fig 2P–2T). Positive dependence of E% on acceptor level (*r = 0*.*474*, Fig 2O) was observed for the 50 ROIs that survive after PFRET analysis, corroborating that this co-localization has no biological significance and those ROIs can be therefore discarded. In the case of APb-FRET analysis of negative control sample, an E% of 7 ± 2% (mean ± SD), ranging from 2 to 12%, was obtained.

FRET controls performed allow us to determine a baseline for positive and specific FRET signal for co-localization. Next, BrU-RNA and microtubules co-localization was analyzed by PFRET. BrU-RNA found in Schwann cell cytoplasm and axoplasm overlaps with microtubule signal with an E% of 74 ± 12% (mean ± SD), ranging from 30 to 90% (Fig 1E and 1F). ROIs where FRET interaction is due to specific encounters between BrU-RNA (donor) and microtubules (acceptor) are shown in Fig 1G (Schwann cell cytoplasm, yellow ROIs) and 1H (axoplasm, yellow ROIs). These ROIs displays E% independent on acceptor (Fig 1I, *r = 0*.*0002*). The positive co-localization found by PFRET between BrU-RNA and microtubules was confirmed by APb-FRET (Fig 1J–1P). E% between BrU-RNA and microtubules by APb-FRET analysis ranges from 5 to 60%, with a mean ± SD of 25 ±16%, and a (very weak negative relation) independence on acceptor level (Fig 1P). Note that the differences in E% means determined by PFRET and APb-FRET approaches for BrU-RNA/ microtubules (49 point) are in accordance with E% difference found by both approaches for Vim/Vim FRET positive control (Fig 1Q).

**Fig 3:**
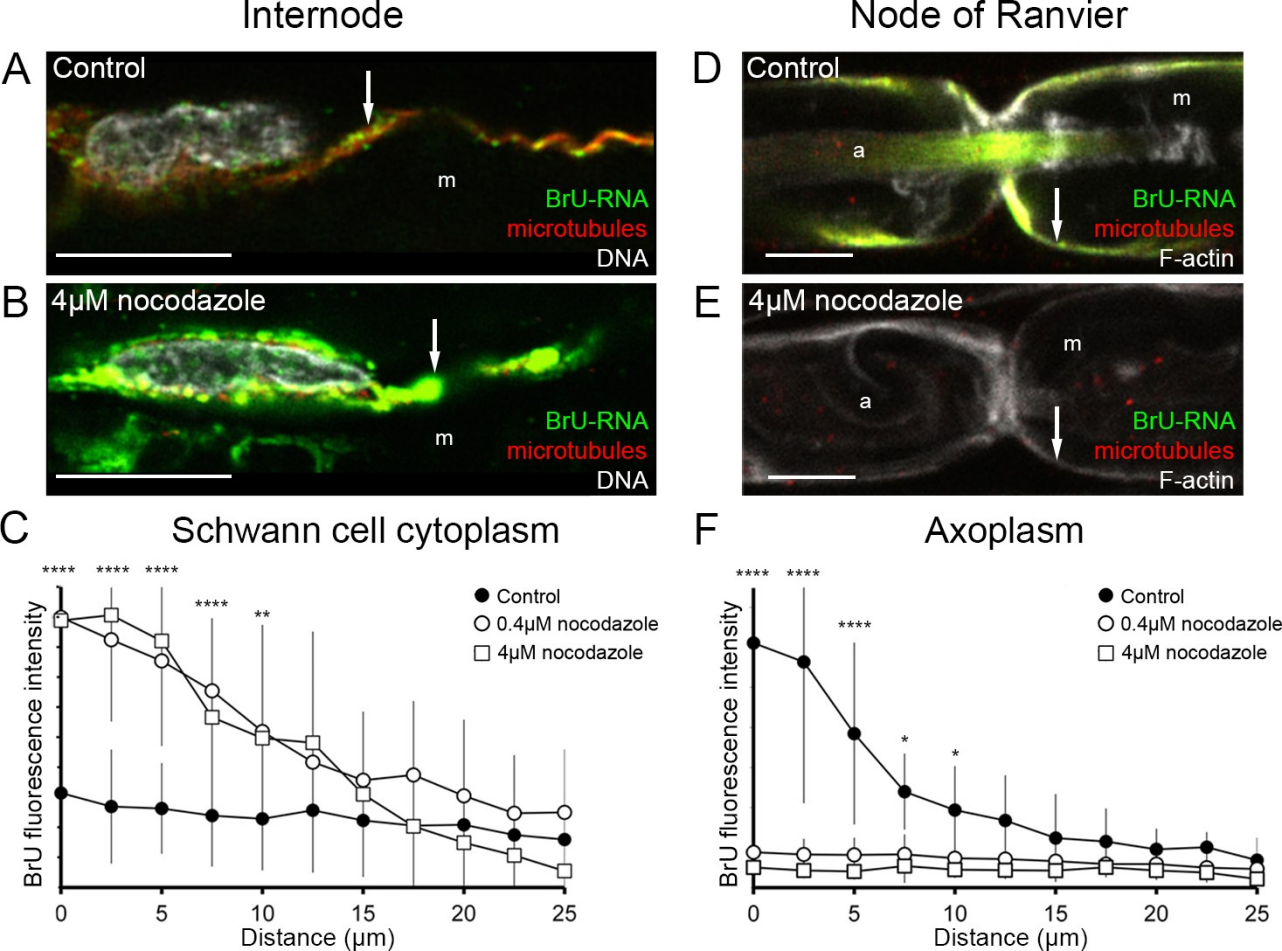
Effect of nocodazole treatment on BrU-RNA levels in Schwann cell cytoplasm and axoplasm from proximal nerve stumps. BrU-RNA fluorescence intensity was measured on single focal planes of fibers incubated in BrU and nocodazole containing media. Nocodazole was used at 0 μM (A, D), 0.4 μM or 4 μM (B, E). BrU-RNA signal is shown in green. DAPI DNA staining (white in A and B) maps the position of nuclei. Phalloidin F-actin staining (white in D and E) delineated the boundaries of Schwann cell and axon. Anti-β-tubulin staining (red) reveals the presence of microtubules in fibers. Bar: 10 μm. m: myelin. a: axon. Arrow: Abaxonal Schwann cell cytoplasm. C) BrU-RNA fluorescence intensity measured on Schwann cell cytoplasm was plotted every 2.5 μm from perinuclear region. F) BrU-RNA fluorescence intensity measured on axoplasm was plotted every 2.5 μm from node of Ranvier. Each value is mean ± SD from at least 10 fibers. P-value code: **** p<0.0001, ** p<0.01, * p<0.05.

To determine if Schwann cell-to-axon RNA transfer is dependent on microtubules, the microtubule network was disrupted by nocodazole (Fig 3). Treatment of fibers with 0.4 or 4 μM nocodazole during the 6 hours of BrU-RNA labeling almost completely disrupts microtubule network (compare red signals in Fig 3A with 3B and Fig 3D with 3E). An apparent accumulation of BrU-RNA signal in Schwann cell abaxonal internodal cytoplasm can be observed in nocodazole-treated fibers (compare green signals in Fig 3A and 3B). Quantification of BrU-RNA fluorescence intensity in Schwann cell cytoplasm 25 μm from perinuclear region, confirmed a higher level of BrU-RNA signals in the first 10 microns comparing nocodazole-treated fibers with control ones (Fig 3C). The opposite effect was observed in Schwann cell cytoplasm (microvilli) and axoplasm at node of Ranvier of nocodazole-treated fibers, where BrU-RNA signal is absent (compare green signals in Fig 3D and 3E). Quantification of BrU-RNA fluorescence intensity in axoplasm at nodes of Ranvier of control fibers (Fig 3F, black circles) showed its typical gradient distribution, with a maximal signal in the nodal region that decreases towards internode. In contrast, axoplasm of nocodazole-treated fibers showed a background level of fluorescence intensity for BrU-RNA signal (Fig 3F, open circles 0.4 μM nocodazole and open squares 4 μM nocodazole). Colchicine treatment of sciatic nerve fibers lead to similar effects (S2A and S2B Fig). The possible RNA synthesis inhibition upon microtubule depolymerization was controlled in fibroblast cultured cells. Nocodazole or colchicine treatment does not reduce the amount of BrU-RNA labeling (S2C Fig). Mechanical resilience and structural integrity of peripheral nerve fibers relies mostly on the Schwann cell basal lamina [37]. However, at high nocodazole concentration (100 μM) treatment peripheral nerve fibers diminish 28% its elasticity [38], associated with the architectural change of the fiber. Although in our work, the nocodazole treatment was less hard (maximal concentration used 4μM) we explore if mislocalization of BrU-RNA after nocodazole treatment could be due to structural collapse of the cells. S2D Fig show that distribution of molecular hallmarks of peripheral nerve fiber morphology [39–42] were unaltered with the nocodazole treatment performed in this work, demonstrating BrU-RNA distribution changes cannot be explain by loss in nerve fiber structure. The results described here indicate that transport of Schwann cell-derived RNAs is dependent on microtubule network integrity, since disruption of microtubules completely interrupts the transfer of BrU-RNA to axons, as well as the transport from Schwann cell nucleus to Schwann cell cytoplasm at nodes of Ranvier (microvilli). Moreover, BrU-RNA accumulates at the Schwann cell perinuclear region.

**Fig 4:**
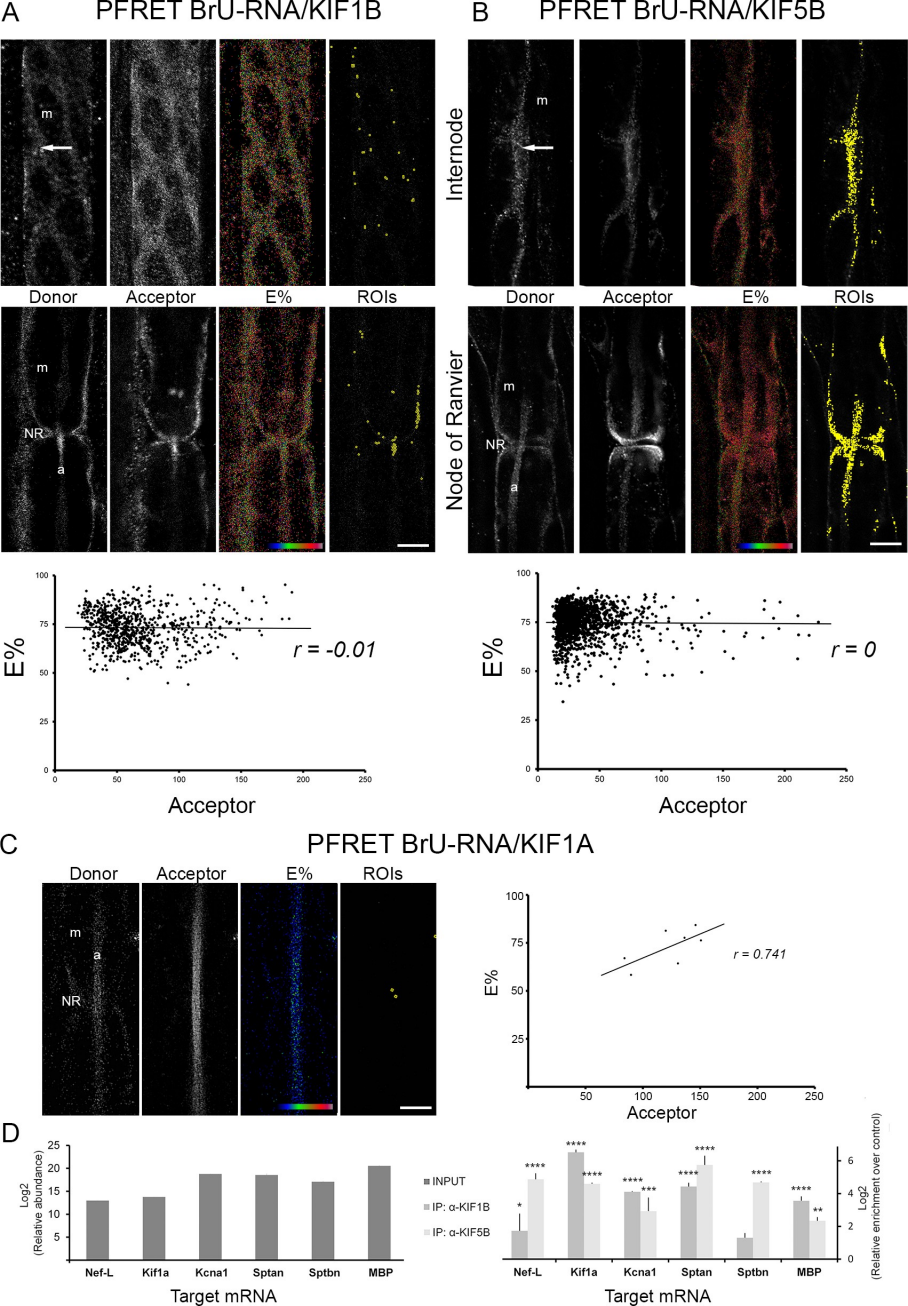
Co-localization analysis between BrU-RNA and KIFs in proximal nerve stumps. A) Representative single focal planes of sciatic nerve fibers at the internode and node of Ranvier are illustrated. Fibers were labeled to detect newly synthesized RNA (BrU-RNA) as donor and KIF1B as acceptor. FRET efficiency (E%, rainbow LUT 0% -black- to 100% -white) at each pixel is indicated. Position on Schwann cell internodal cytoplasm and axoplasm of ROIs having specific PFRET signal is also shown. E% versus acceptor level is plotted for ROIs having specific PFRET signal (*r = -0*.*01*). B) Representative single focal planes of sciatic nerve fibers at the internode and node of Ranvier are depicted. Fibers were labeled to visualize newly synthesized RNA (BrU-RNA) as donor and KIF5B as acceptor. FRET efficiency (E%, rainbow LUT 0% -black- to 100% -white) at each pixel is indicated. Position on Schwann cell internodal cytoplasm and axoplasm of ROIs having specific PFRET signal is also presented. E% versus acceptor level is plotted for ROIs having specific PFRET signal (*r = 0*). C) Representative single focal plane of sciatic nerve fibers at the node of Ranvier are shown. Fibers were labeled to visualize newly synthesized RNA (BrU-RNA) as donor and KIF1A as acceptor. FRET efficiency (E%, rainbow LUT 0% -black- to 100% -white) at each pixel is indicated. Position of ROIs obtained after PFRET analysis is also presented. E% versus acceptor level is plotted for those ROIs (*r = 0*.*741*). Bar size: 20 μm. NR: node of Ranvier. m: myelin. a: axon. Arrows: Cajal Bands. D) qPCR of mRNA isolated from proximal nerve stumps ribonucleoparticles (Input) or its immunoprecipitation using anti-KIF1B or anti-KIF5B antibodies. Relative abundance (log2) and relative enrichment (log2) of immunoprecipitations over the control condition of target mRNAs were plotted (mean ± SD) (see text). P-value code: **** p<0.0001, *** p<0.001, ** p<0.01, * p<0.05.

Microtubule-based RNA transport towards the cell periphery involves kinesin-mediated movement. KIF1B has been reported as a molecular motor for mRNAs in oligodendrocytes [13,14,43]. We detected KIF1B in both Schwann cells (Fig 4A, acceptor) and axon (Fig 4A, acceptor), where it co-localizes with BrU-RNA signal (Fig 4A, donor). FRET efficiency of BrU-RNA-KIF1B co-localization (Fig 4A, E%) showed an independence on acceptor levels (Fig 4A, graphic, r = -0.01) for ROIs located in Schwann cell cytoplasm, and also in axoplasm (Fig 4A, ROIs). Additionally, KIF5 has been mentioned as RNA transport motors in neuronal processes [15,16,44]. FRET co-localization between BrU-RNA (Fig 4B, donor) and KIF5B (Fig 4B, acceptor) signals were observed in the Schwann cell cytoplasm at the internodes and nodes of Ranvier and in axoplasm at nodes of Ranvier, with a FRET efficiency ranging from 40 to 80% (Fig 4B, E%). Pearson’s correlation analysis between E% and acceptor levels, indicates that ROIs showing clustered FRET behavior (Fig 4B, graphic, r = 0) are located in all mentioned subcellular locations (Fig 4B, ROIs). To determine the specificity of FRET analysis in our system, we performed confocal FRET microscopy with KIF1A, a neuronal specific kinesin family member which has not been reported to transport RNAs [45–47]. KIF1A signal was observed specifically at the axoplasm in sciatic nerve fibers (Fig 4C, acceptor). FRET analysis of co-localization between BrU-RNA (Fig 4C, donor) and KIF1A gave negative results (observe the blue color of E% LUT image, Fig 4C, E%). In addition, no ROIs for specific interaction events between BrU-RNA and KIF1A signal were obtained, since the few ROIs (Fig 4C, ROIs) that return after PFRET analysis showed a strong positive dependence on acceptor level (Fig 4C, graphic, r = 0.741) and were then discarded.

Our next goal was to obtain a list of mRNA candidates to be transferred from Schwann cell to axon. For this, we performed RNAseq screening of BrU-RNA immunoprecipitated from proximal nerve stumps incubated in BrU-containing media. mRNA transcribed in the Schwann cell nucleus but encoding proteins uniquely detected in neurons are candidates to be transferred. As expected, we found genes known to be highly expressed in Schwann cells leading our list (S1 Table, G marker). In addition, among the 7410 genes detected in the BrU-RNA sample we found several mRNAs candidates to be transferred (S1 Table, N marker). The exclusive axonal location of proteins encoded by the candidate mRNAs in our proximal nerve stump model, was corroborated by confocal immunohistochemistry for NEF-L and KIF1A (S3A and S3B Fig, respectively). To confirm the association of KIF1B and KIF5B with mRNAs candidates to be transferred from Schwann cells to axons (*Nef-L*, *Kif1a* and *Kcna1*), we performed quantitative RT-PCR of mRNAs co-immunoprecipitated using anti-KIF1B or anti-KIF5B antibodies (Fig 4D). All mRNAs candidates tested are in association with KIF1B and KIF5B in proximal sciatic nerve stumps (Fig 4D, IP: a-KIF1B and IP: a-KIF5B). Additionally, we selected cytoskeletal proteins spectrin αII (*Sptan1*) and spectrin βII (*Sptbn1*) (S1 Table) known to be enriched at node of Ranvier [48]. *Sptan1* mRNA was present in the input sample and co-immunoprecipitated with KIF1B and KIF5B, meanwhile *Sptbn1* was found in the anti-KIF5B immunoprecipitation (Fig 4D). As a positive control of qPCR experiments *MBP* (Myelin Basic Protein) mRNA, previously reported to be associated with KIF1B in central nervous system glia [14], was chosen. *MBP* mRNA was present in RNAs purified from Input sample, and in anti-KIF1B immunoprecipitated one (Fig 4D). Surprisingly, *MBP* mRNA was also present in anti-KIF5B isolated sample (Fig 4D), an unreported finding in peripheral glial cells. Based on our findings, mRNAs codifying axonal-specific proteins are among Schwann cell newly synthesized RNAs. In addition, some of them are associated with KIF1B and KIF5B microtubules-based motors in sciatic nerve fibers.

## Discussion

Macromolecular transference from Schwann cells to axons have been reported for RNA, ribosomes and proteins [49,50]. In recent years, our group has been investigating the transfer of RNA molecules from Schwann cells to axons in rat sciatic nerve [4,5]. RNA transfer is assessed by incubating a segment of sciatic nerve in culture media that contains 5-bromouridine (BrU) [4,5,17], which is co-transcriptionally incorporated to the nascent RNA. Immunohistochemical analysis of BrU-incubated nerve segments with anti-BrU antibodies revealed axonal presence of BrU-RNA. Since in our experiments the axons in the nerve segment are separated from the cell body, the only possible nucleus of BrU-RNA origin is the Schwann cell nucleus. Nuclear origin of BrU-RNA was demonstrated by RNA polymerase II inhibition experiments [5]. Additionally, in the same article we demonstrated that RNA transference is a physiological phenomenon that increases under injured conditions [5], making the sciatic nerve section a very useful model to visualize this phenomenon. Performing BrU pulse-chase experiments we previously estimated newly-synthesized RNA transport velocity inside Schwann cell cytoplasm in accordance with microtubule-based transport [4]. In this work, we tested the dependence of Schwann-axon BrU-RNA transfer on microtubule network.

Here we demonstrated that BrU-RNA co-localizes with microtubules in nerve fibers (Fig 1). Immunohistochemical analyses of this co-localization were performed with a modified protocol from our previous works [4,5] which includes the extraction of monomeric tubulin, whose bright diffuse staining would otherwise obscure microtubule visualization. Tubulin extraction, which is achieved by permeabilization before fixation, does not affect BrU-RNA labeling (S1C–S1F Fig). In addition, we demonstrated that BrU-RNA transport from Schwann cell nucleus to nodal microvilli is dependent on microtubules. Its disruption with nocodazole or colchicine decreases BrU-RNA signal in nodal microvilli accompanied by an increase in abaxonal internodal cytoplasm (perinuclear region) signal (Fig 3 and S2A and S2B Fig), consistent with inhibition of RNA transport, not synthesis (S2C Fig). In fact, colchicine has no effect on overall transcription; meanwhile with nocodazole an overall increase in gene expression was observed. Gene expression changes resulting from changes in microtubule dynamics (by depolymerization or stabilization of microtubules) has been previously reported [51–53]. For example, it has been observed that the use of nocodazole could activate transcription throughout the NF-kB pathway [51,53].

Since the transport of BrU-RNA from Schwann cell nucleus to cytoplasm at node of Ranvier (microvilli) is carried out through microtubules, we ask which kinesin might be involved in this action. Our results showed a co-localization between BrU-RNA and KIF1B or KIF5B (Fig 4A and 4B), two kinesins reported to act as molecular motors of RNA-containing particles in the nervous system [13–16,43,44]. However, no co-localization was observed with a KIF (Fig 4C) involved in the transport of membrane-based organelles [45–47]. Co-localization results are usually confirmed by immunoprecipitation, which in our case would imply the demonstration of BrU-RNA in anti-KIF immunoprecipitated ribonucleoparticles obtained from axoplasm purificated fractions. Experiments carried out to show this gave no results (data not shown), probably due to limitations in the sensitivity of techniques in relation with the axoplasm yield obtained from proximal nerve stumps. Using an alternative approach we determined the presence of the mRNA encoding the neurofilament light chain (*Nef-L*) in KIF5B and KIF1B immunoprecipitated ribonucleoparticles (Fig 4D), and we confirmed its presence in BrU-labeled Schwann cell derived RNAs (S1 Table). mRNA encoding the neurofilament light chain (*Nef-L*) is the only mRNA previously identified as transferred from Schwann cells to axons [5]. In addition, the neuron-specific kinesin superfamily member *kif1a* mRNA [54] was also found in BrU-labeled Schwann cell derived RNAs (S1 Table) and associated with the KIF1B and KIF5B molecular motors (Fig 4D). The axonal specific expression of NEF-L and KIF1A proteins was confirmed for our proximal stump experimental model (S3 Fig). Altogether, our results suggest that the kinesin 1 and 3 superfamily proteins KIF5B and KIF1B could carry out Schwann cell-to-axon transferred-RNA travel inside the Schwann cell cytoplasm.

We propose a model in which RNAs are transcribed in the Schwann cell nucleus and transported to Schwann cell microvilli at nodes of Ranvier by microtubules and kinesins. At this point, Schwann cell newly-synthesized RNAs probably translocate from the microtubule network to microfilaments. Our previous work [4,5] has demonstrated that the actin cytoskeleton and its associated molecular motor myosin Va, are necessary to cross the double membrane boundary between Schwann cell and axons at nodes of Ranvier. The complex mechanism through which newly-synthesized RNAs cross the cytoplasmic membranes of both cells is presently unknown, but probably involve tunneling nanotubes [17]. Whether microtubules are the means by which Schwann cell-derived RNAs travel inside axons remains to be elucidated.

## Supporting information

S1 Fig A) Representative single focal plane of abaxonal Schwann cell cytoplasm at the node of Ranvier (NR), showing the characteristic distribution of cytoplasm in channel-like structures knowing as Cajal bands (arrows) depicted by labeling of F-actin (white). Myelin, completely depleted of cytoplasm is showed by Nile red lipid labeling (red). B) Representative single focal plane of central region of a nerve fiber at the node of Ranvier, showing a lateral view of abaxonal Schwann cell cytoplasm and axoplasm (a) depicted by F-actin labeling. Note the absence of F-actin signal in myelin (m). The characteristic paranodal loops (asterisk) and microvilli (arrow) of the nodal region are indicated. C-D) Negative controls of BrU-labeling in proximal nerve stumps. Representative single focal planes of sciatic nerve fibers at node of Ranvier are illustrated. C) Fibers were incubated in BrU-containing media and then treated for immunohistochemistry to visualize BrU-RNA (green). D) Fibers were incubated in medium without BrU and then treated for immunohistochemistry as in (C). E) Fibers were incubated in BrU-containing media and then treated for immunohistochemistry, omitting the primary anti-BrU antibody. F) Fibers were incubated in BrU-containing media. Tissue RNAse digestion was performed and fibers were then treated for immunohistochemistry to detect BrU-RNA. Phalloidin F-actin staining (white) defines the shape of Schwann cell and axon. NR: node of Ranvier. m: myelin. a: axon. Bar: 10 μm.
(TIF)

S2 Fig A and B) Effect of colchicine treatment on BrU-RNA levels in Schwann cell cytoplasm and axoplasm from proximal nerve stumps. BrU-RNA fluorescence intensity was measured on single focal planes of fibers incubated in BrU and colchicine containing media. Colchicine was used at 0 μM or 5 μM. A) BrU-RNA fluorescence intensity measured on Schwann cell cytoplasm was plotted every 2.5 μm from perinuclear region. B) BrU-RNA fluorescence intensity measured on axoplasm was plotted every 2.5 μm from node of Ranvier. Each value is mean ± SD from at least 10 fibers. C) BrU-RNA fluorescence intensity was measured on single focal planes of 3T3-L1 fibroblast incubated in BrU and 0.4 μM nocodazole or BrU and 5 μM colchicine containing media and compared with BrU-RNA fluorescence intensity from 3T3-L1 fibroblast incubated in BrU containing media (control). Graphic represents mean ± SD of total BrU-RNA fluorescence intensity in each cell/cell area. Insets shows representative images of each condition. P-value code: **** p<0.0001, *** p<0.001, ** p<0.01, *p<0.05, n.s. not significant. D) Molecular organization of control and nocodazole-treated sciatic nerve fibers. Representative single focal plane of nerve fibers in the internode at adaxonal cytoplasm and node of Ranvier at the axonal level are shown stained to visualize microtubules (a, a’, e and e’), F-actin (b, b’, f and f’), vimentin (c, c’), MAG (d and d’) or Caspr2 (g, g’). Note that microtubules and F-actin are showed for the same fiber in each condition. Bar size: 10 μm. m: myelin. a: axon. Arrow: Cajal band. Arrowhead: Schmidt-Lanterman Incisure.
(TIF)

S3 Fig Representative single focal plane of nerve fibers at the node of Ranvier at the axonal level, immunostained to visualize NEF-L (A, red) or KIF1A (B, red). Phalloidin F-actin staining (white) defines the shape of Schwann cell and axon. NR: node of Ranvier. m: myelin. a: axon. Bar: 10 μm.
(TIF)

S1 Table Expression levels of selected BrU-labeled Schwann cell RNAs obtained from BrU-RNA immunoprecipitation and RNA-seq experiments. TPM: Transcript per million, G: Glial marker, N: neuronal marker. (See text).
(DOCX)
