# Supplementary figures and images for: Association of microtubules and axonal RNA transferred from myelinating Schwann cells in rat sciatic nerve
Source: PLoS One. 2020 May 29;15(5):e0233651. doi: 10.1371/journal.pone.0233651 (PMC7259579; doi:10.1371/journal.pone.0233651)

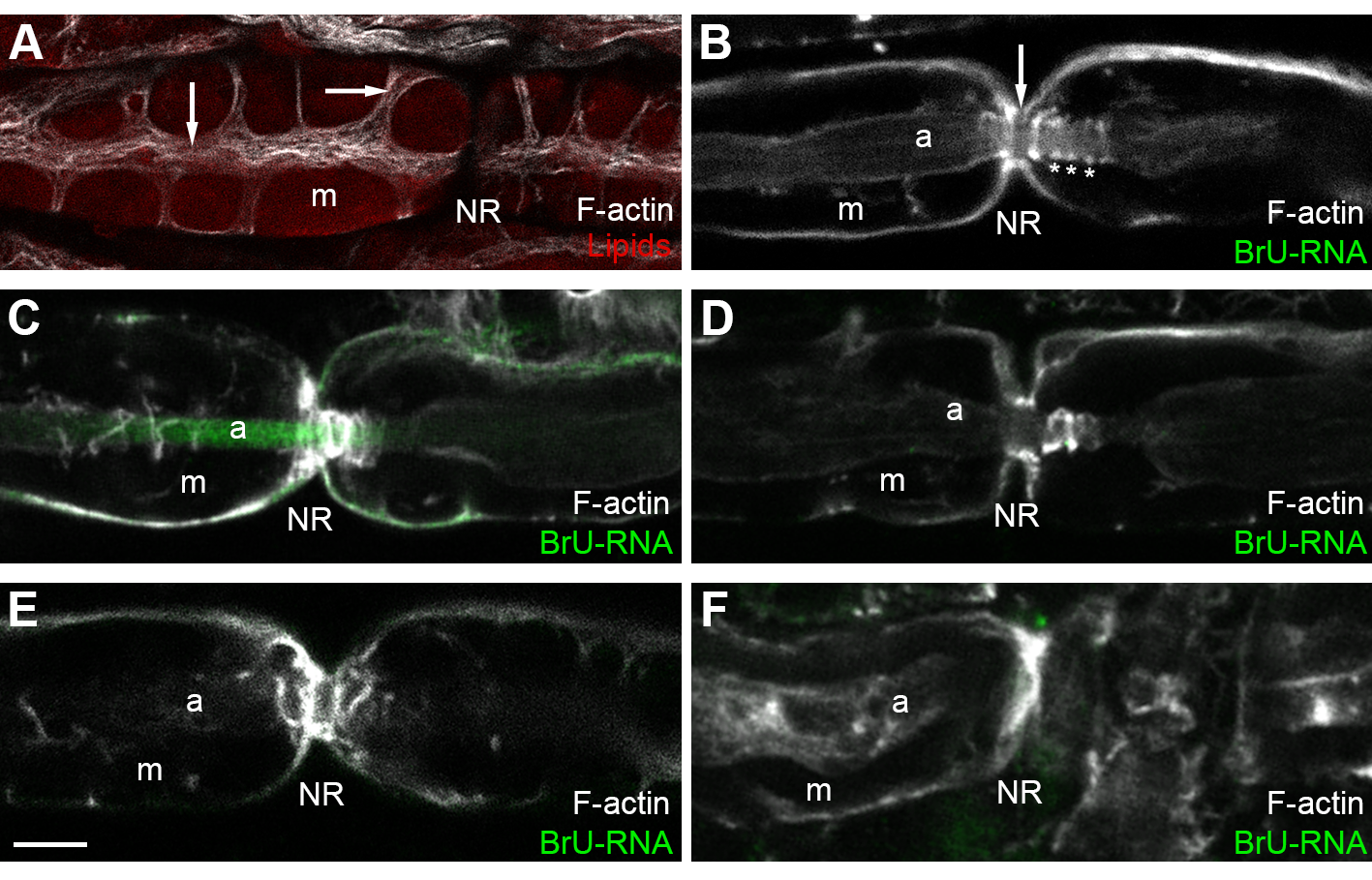

Supplement: S1 Fig — A) Representative single focal plane of abaxonal Schwann cell cytoplasm at the node of Ranvier (NR), showing the characteristic distribution of cytoplasm in channel-like structures knowing as Cajal bands (arrows) depicted by labeling of F-actin (white). Myelin, completely depleted of cytoplasm is showed by Nile red lipid labeling (red). B) Representative single focal plane of central region of a nerve fiber at the node of Ranvier, showing a lateral view of abaxonal Schwann cell cytoplasm and axoplasm (a) depicted by F-actin labeling. Note the absence of F-actin signal in myelin (m). The characteristic paranodal loops (asterisk) and microvilli (arrow) of the nodal region are indicated. C-D) Negative controls of BrU-labeling in proximal nerve stumps. Representative single focal planes of sciatic nerve fibers at node of Ranvier are illustrated. C) Fibers were incubated in BrU-containing media and then treated for immunohistochemistry to visualize BrU-RNA (green). D) Fibers were incubated in medium without BrU and then treated for immunohistochemistry as in (C). E) Fibers were incubated in BrU-containing media and then treated for immunohistochemistry, omitting the primary anti-BrU antibody. F) Fibers were incubated in BrU-containing media. Tissue RNAse digestion was performed and fibers were then treated for immunohistochemistry to detect BrU-RNA. Phalloidin F-actin staining (white) defines the shape of Schwann cell and axon. NR: node of Ranvier. m: myelin. a: axon. Bar: 10 μm. (TIF) [file pone.0233651.s001.tif]

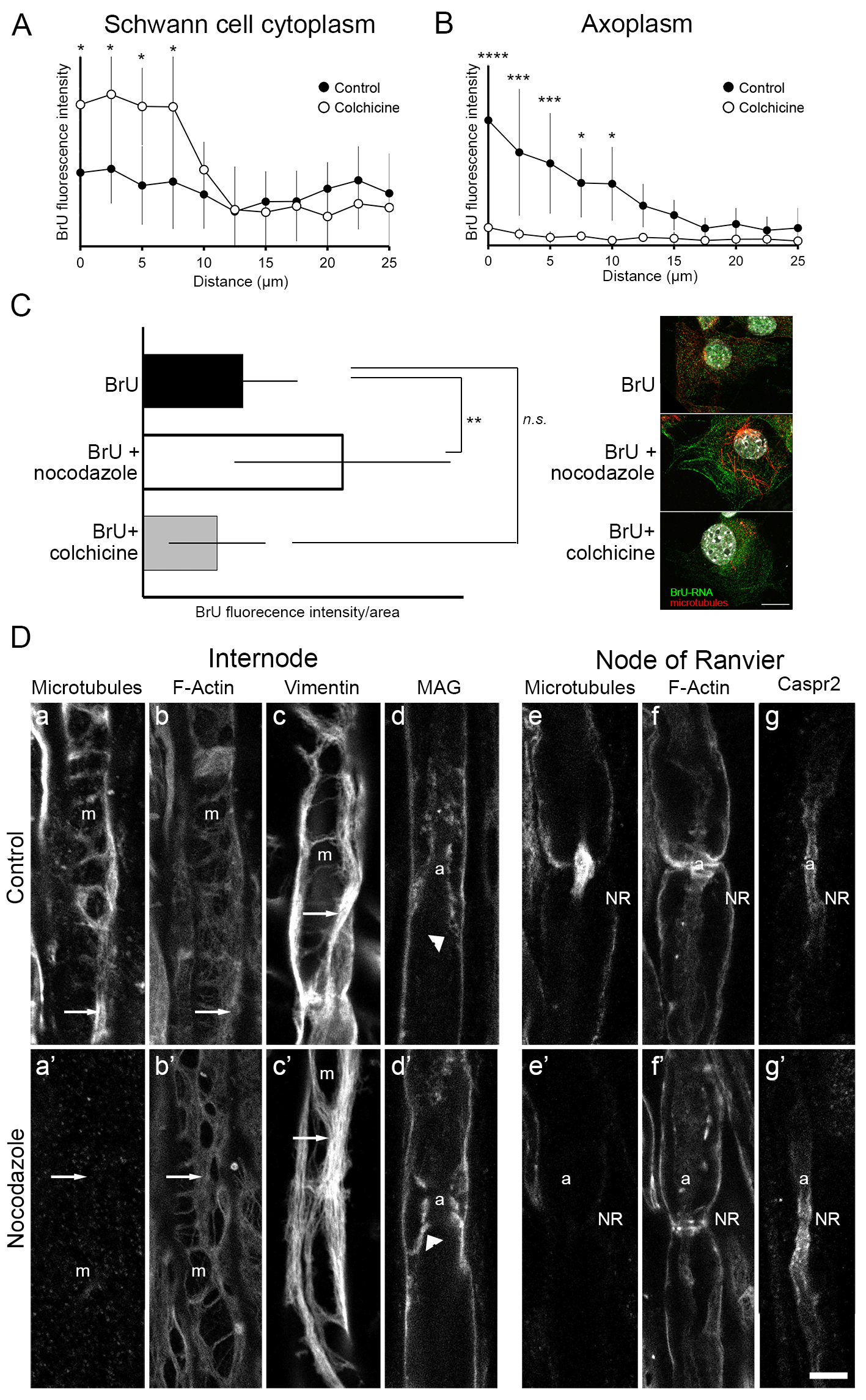

Supplement: S2 Fig — A and B) Effect of colchicine treatment on BrU-RNA levels in Schwann cell cytoplasm and axoplasm from proximal nerve stumps. BrU-RNA fluorescence intensity was measured on single focal planes of fibers incubated in BrU and colchicine containing media. Colchicine was used at 0 μM or 5 μM. A) BrU-RNA fluorescence intensity measured on Schwann cell cytoplasm was plotted every 2.5 μm from perinuclear region. B) BrU-RNA fluorescence intensity measured on axoplasm was plotted every 2.5 μm from node of Ranvier. Each value is mean ± SD from at least 10 fibers. C) BrU-RNA fluorescence intensity was measured on single focal planes of 3T3-L1 fibroblast incubated in BrU and 0.4 μM nocodazole or BrU and 5 μM colchicine containing media and compared with BrU-RNA fluorescence intensity from 3T3-L1 fibroblast incubated in BrU containing media (control). Graphic represents mean ± SD of total BrU-RNA fluorescence intensity in each cell/cell area. Insets shows representative images of each condition. P-value code: **** p<0.0001, *** p<0.001, ** p<0.01, *p<0.05, n.s. not significant. D) Molecular organization of control and nocodazole-treated sciatic nerve fibers. Representative single focal plane of nerve fibers in the internode at adaxonal cytoplasm and node of Ranvier at the axonal level are shown stained to visualize microtubules (a, a’, e and e’), F-actin (b, b’, f and f’), vimentin (c, c’), MAG (d and d’) or Caspr2 (g, g’). Note that microtubules and F-actin are showed for the same fiber in each condition. Bar size: 10 μm. m: myelin. a: axon. Arrow: Cajal band. Arrowhead: Schmidt-Lanterman Incisure. (TIF) [file pone.0233651.s002.tif]

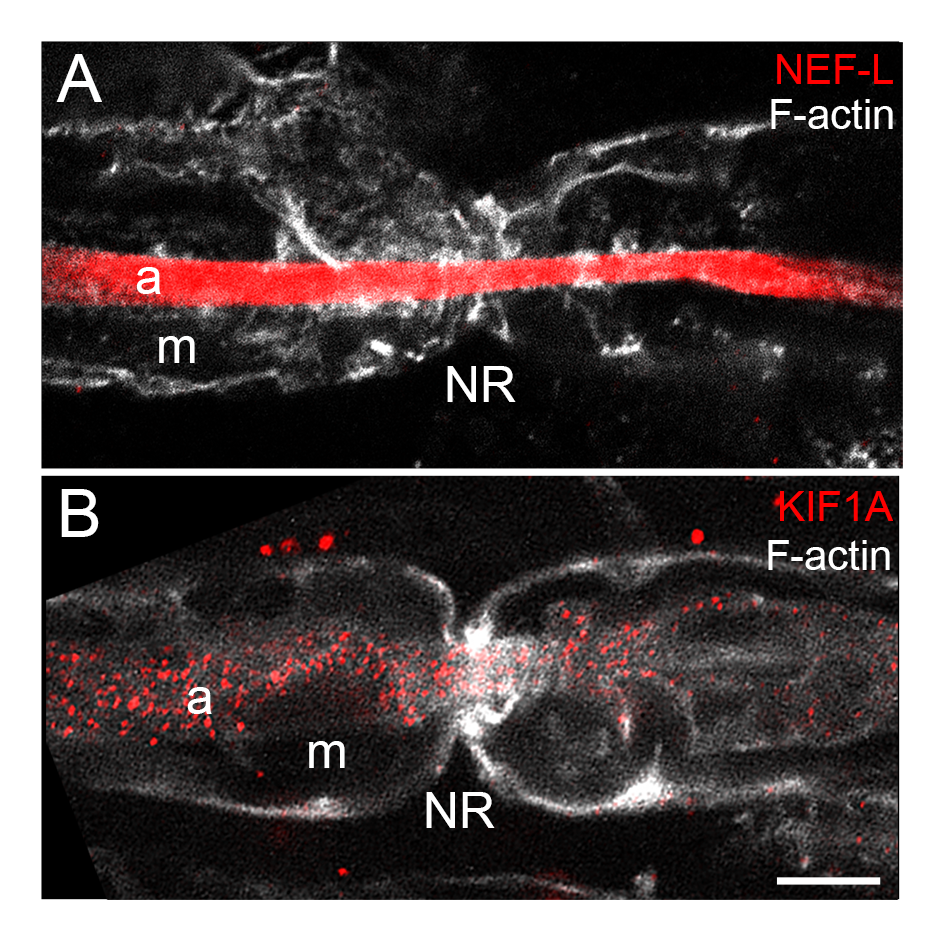

Supplement: S3 Fig — Representative single focal plane of nerve fibers at the node of Ranvier at the axonal level, immunostained to visualize NEF-L (A, red) or KIF1A (B, red). Phalloidin F-actin staining (white) defines the shape of Schwann cell and axon. NR: node of Ranvier. m: myelin. a: axon. Bar: 10 μm. (TIF) [file pone.0233651.s003.tif]
