# Supplementary material for: Association of microtubules and axonal RNA transferred from myelinating Schwann cells in rat sciatic nerve
Source: PLoS One. 2020 May 29;15(5):e0233651. doi: 10.1371/journal.pone.0233651 (PMC7259579; doi:10.1371/journal.pone.0233651)
Supplement: S1 Table — TPM: Transcript per million, G: Glial marker, N: neuronal marker. (See text). (DOCX) [file pone.0233651.s004.docx]

**S1 Table. Expression levels of selected BrU-labeled Schwann cell RNAs obtained from BrU-RNA immunoprecipitation and RNA-seq experiments.** TPM: Transcript per million, G: Glial marker, N: neuronal marker. (See text).
